# Metal Transformation by a Novel *Pelosinus* Isolate From a Subsurface Environment

**DOI:** 10.3389/fmicb.2018.01689

**Published:** 2018-08-17

**Authors:** Allison E. Ray, Stephanie A. Connon, Andrew L. Neal, Yoshiko Fujita, David E. Cummings, Jani C. Ingram, Timothy S. Magnuson

**Affiliations:** ^1^Department of Biological Sciences, Idaho State University, Pocatello, ID, United States; ^2^Idaho National Laboratory, Idaho Falls, ID, United States; ^3^California Institute of Technology, Pasadena, CA, United States; ^4^Savannah River Ecology Laboratory, University of Georgia, Aiken, SC, United States; ^5^Department of Biology, Point Loma Nazarene University, San Diego, CA, United States

**Keywords:** toxic metal reduction, bioremediation, subsurface environment, fermentative bacterium, *Pelosinus*

## Abstract

The capability of microorganisms to alter metal speciation offers potential for the development of new strategies for immobilization of toxic metals in the environment. A metal-reducing microbe, “*Pelosinus lilae*” strain UFO1, was isolated under strictly anaerobic conditions from an Fe(III)-reducing enrichment established with uncontaminated soil from the Department of Energy Oak Ridge Field Research Center, Tennessee. “*P. lilae*” UFO1 is a rod-shaped, spore-forming, and Gram-variable anaerobe with a fermentative metabolism. It is capable of reducing the humic acid analog anthraquinone-2,6-disulfonate (AQDS) using a variety of fermentable substrates and H_2_. Reduction of Fe(III)-nitrilotriacetic acid occurred in the presence of lactate as carbon and electron donor. Ferrihydrite was not reduced in the absence of AQDS. Nearly complete reduction of 1, 3, and 5 ppm Cr(VI) occurred within 24 h in suspensions containing 10^8^ cells mL^−1^ when provided with 10 mM lactate; when 1 mM AQDS was added, 3 and 5 ppm Cr(VI) were reduced to 0.1 ppm within 2 h. Strain UFO1 is a novel species within the bacterial genus *Pelosinus*, having 98.16% 16S rRNA gene sequence similarity with the most closely related described species, *Pelosinus fermentans* R7^T^. The G+C content of the genomic DNA was 38 mol%, and DNA-DNA hybridization of “*P. lilae*” UFO1 against *P. fermentans* R7^T^ indicated an average 16.8% DNA-DNA similarity. The unique phylogenetic, physiologic, and metal-transforming characteristics of “*P. lilae*” UFO1 reveal it is a novel isolate of the described genus *Pelosinus*.

## Introduction

The pollution of groundwater by metal and radionuclide contaminants continues to pose a clear threat to public health (EPA, 2003), and remediation of subsurface environments is a growing environmental and economic challenge (White et al., 1997; NABIR, 2003). In the United States, the Department of Energy (DOE) has generated 6.4 trillion L of contaminated groundwater in 5700 distinct plumes and 40 million m^3^ of contaminated soil and subsurface environs as a result of Cold War era nuclear weapons production (USDOE, 1997). After petroleum fuel and chlorinated hydrocarbons, metals and radionuclides (lead, chromium, arsenic, uranium, strontium-90, and tritium) represent the most common contaminants found in soils and groundwater at DOE facilities (Riley et al., 1992). Moreover, metallic and radioactive contaminants cannot be biodegraded and often prove to be the most challenging of hazardous wastes at DOE sites (NABIR, 2003).

In contrast to organic contaminants that may be completely oxidized to carbon dioxide and water, remediation of metals is more problematic, owing to the fact that the metals cannot be destroyed, and changes in their oxidation or reduction potential often dictate their ultimate environmental fate (Lovley and Lloyd, 2000). Generally, the goal of metal remediation is to limit contaminant mobility. The potential for immobilization of a given metal in the environment is principally dependent upon its chemical speciation. Thus, the aim is to either induce or maintain chemical conditions that result in metal species with reduced mobility. Depending on the ambient redox conditions, many toxic metals and radionuclides can be highly soluble, and thus mobile, in groundwater (Lovley, 2001). Microorganisms are capable of altering chemical speciation via redox reactions, thereby influencing solubility, transport properties, and bioavailability of metallic contaminants in subsurface environments. For example, bioreduction of highly soluble Cr(VI) and U(VI) can result in conversion to insoluble species [e.g., Cr(III) and U(IV)] that precipitate from solution (Tabak et al., 2005).

Microbial Fe(III) reduction is an important environmental process, and many Fe(III)-reducing bacteria have been found to also reduce high-valence metal contaminants (Lloyd, 2003). In addition, Fe(III)-reducing bacteria have been shown to utilize humic acids and synthetic electron-shuttling moieties, such as anthraquinone-2,6-disulfonate (AQDS), as electron acceptors (Lovley et al., 1996; Scott et al., 1998), which in turn can mediate the indirect reduction of Fe(III) and other metals (Lovley et al., 1998; Fredrickson et al., 2000; Nevin and Lovley, 2000). The physiologic diversity of electron transport to ferruginous mineral substrates are distributed into distinct groups of Fe(III)-reducing microorganisms. One group consists of the respiratory Fe(III)-reducers, in which reduction occurs via an electron transfer event coupled to energy generation. Representatives of this group include well-characterized organisms such as *Geobacter* spp., and *Shewanella* spp. (Lovley et al., 1987, 1989, 1993; Caccavo et al., 1992, 1994, 1996). The second group includes fermentative microorganisms that use humic acids and Fe(III)-bearing minerals as an electron sink for excess reducing power formed during fermentative metabolism (Benz et al., 1998; Borch et al., 2005; Shelobolina et al., 2007). Although these organisms have been detected in a variety of habitats where Fe(III) reduction is an important ecophysiological process (Kappler et al., 2004), this group is less well understood physiologically and phylogenetically. Recent studies on isolated members of the genus *Pelosinus* reveals novel mechanisms for metal sequestration and transformation (Beller et al., 2013; Thorgersen et al., 2017), and exhibit unique phylogenomic traits such as multiple, distinct copies of 16S rRNA genes (Ray et al., 2010), potentially confounding phylogenetic analysis of this group common in subsurface environments. Thus, examining new representatives of this group contributes to a broader understanding of the organisms involved in the biotransformation of metals in subsurface environments.

*In situ* biological treatment can be less expensive and less disruptive than traditional *ex situ* technologies for remediation of metal-contaminated sites, as it relies on indigenous microorganisms to achieve clean-up of hazardous wastes (NABIR, 2003). Examination of the biological potential for metal reduction among native microorganisms is important for implementation of successful remediation strategies. However, little is known about the potential for fermentative, Fe(III)-reducing subsurface microorganisms to play a role in metal bioremediation. Our goal was to isolate and characterize one such organism from a field study area adjacent to a metals-contaminated environment and evaluate its potential for metal bioremediation. Here, we describe “*Pelosinus lilae*” UFO1, isolated from the background or “pristine” area at the Oak Ridge Field Research Center (ORFRC), Tennessee. The unique phylogenetic and metal-transforming characteristics of “*P. lilae*” UFO1 reveal it is a novel species of the described genus designated *Pelosinus*.

## Materials and Methods

### Enrichment and Isolation of Strain UFO1

Strict anaerobic techniques were used throughout this study (Miller and Wolin, 1974; Balch and Wolfe, 1976). Sediment cores from the Field Research Center in Oak Ridge, TN, United States were taken from background well 330, section 02-22 (FWB 330-02-22), at a depth of 0.61–1.12 m below the surface. The groundwater pH was 6.13. Samples were shipped and stored in Mason jars under N_2_ and processed in an anaerobic glove bag under N_2_-CO_2_-H_2_ (75:20:5).

Enrichment cultures were initiated in 27 mL anaerobic pressure tubes (Bellco Glass) containing 9 mL anaerobic freshwater medium (ATCC medium 2129) and ∼1 g FRC sediment. A suspension of 2-line ferrihydrite (∼1 M) was synthesized by dissolving 40 g Fe(NO_3_)_3_⋅9H_2_O in 0.5 L water and adjusting to pH 7.0 with ∼345 mL 1 M KOH, and the resulting precipitate was washed thoroughly in DI water (Schwertmann and Cornell, 2000). The ferrihydrite was used as the terminal electron acceptor for enrichment cultures. Ferrihydrite (∼40 mM) was added to the tubes along with 10 mM acetate, and the tubes were incubated at room temperature. After 9 months, a 30 mM bicarbonate-buffered freshwater medium with 10 mM lactate, 5 mM AQDS as described by Finneran et al. (2002), and ∼20 mM ferrihydrite was used to prepare a dilution series from the iron-reducing culture. Aliquots were removed from the highest dilutions appearing positive for AQDS- and Fe(III)-reduction and streaked on plates of R2A agar (BD Difco™, Franklin Lakes, NJ, United States) supplemented with 20 mM fumarate in an anaerobic chamber. Distinct colonies were picked and re-streaked at least three times prior to transfer to liquid medium.

### Routine Cultivation

After isolation, “*P. lilae*” UFO1 was routinely cultured in anoxic R2A broth and incubated at 30°C. R2A broth was prepared from a dry mix (BD Diagnostic Systems, Franklin Lakes, NJ, United States) or as follows (per liter): 0.5 g yeast extract; 0.5 g proteose peptone; 0.5 g casamino acids; 0.5 g glucose; 0.5 g soluble starch; 0.3 g sodium pyruvate; 0.3 g K_2_HPO_4_; 0.05 g MgSO_4_ (Reasoner and Geldreich, 1985), supplemented with 20 mM fumarate, and adjusted to pH 7. The R2A broth was boiled and cooled under a headspace of N_2_, dispensed into anaerobic pressure tubes or serum vials with a headspace of N_2_, sealed with thick butyl-rubber stoppers, and autoclaved. The effects of temperature, pH, and O_2_ on growth rates were evaluated using R2A broth (adjusted to acidic and alkaline pH where necessary).

Anaerobic, bicarbonate-buffered, freshwater (FW) medium was prepared, as described previously by Finneran et al. (2002) with or without 5 mM AQDS, and dispensed into anaerobic pressure tubes or serum vials under N_2_:CO_2_ (80:20). Tubes or vials were sealed with thick butyl-rubber stoppers and sterilized by autoclaving. FW medium was used for experiments examining electron donor and acceptor utilization.

### Electron Donor and Acceptor Utilization Studies

Cells were harvested by centrifugation from cultures grown in R2A broth, washed twice in anoxic FW medium, and suspended in sterile, pH 7, anoxic FW medium. For screening of potential electron donors and acceptors, sterile anoxic stock solutions were prepared for a variety of electron donors and acceptors and added to the FW medium. Utilization was evaluated in triplicate incubations.

The ability of “*P. lilae*” UFO1 to reduce the following electron acceptors was examined: Fe(III)-NTA, 2 line-ferrihydrite, AQDS, Cr(VI), As(V), NO_3_^-^, NO_2_^-^, SO_4_^2-^, and SeO_4_^2-^. The ability of washed cell suspensions to reduce Cr(VI) was examined under non-growth conditions, defined here by the omission of phosphate from the FW medium. Washed cells were added to the medium to give a concentration of 10^8^ cells mL^-1^.

Carbon substrate utilization by strain UFO1 was examined under anaerobic conditions using the BIOLOG^®^ AN MicroPlate™ (Hayward, CA, United States) (Bochner, 1989). Anaerobic MicroPlates test the ability of a microorganism to utilize or oxidize an array of carbon sources under anaerobic conditions using an artificial tetrazolium colorimetric electron acceptor. A culture of strain UFO1 was grown for 24 h on R2A broth, pelleted and washed in anoxic FW medium, and suspended in the inoculating medium according to the manufacturer’s instructions. Microplates were inoculated in triplicate and incubated at 35°C for 24 h; wells exhibiting a color change due to the reduction of the tetrazolium dye as compared to the negative control (no carbon source) were considered positive for substrate utilization.

Potentially fermentable substrates were examined in FW medium (containing 0.5 mM cysteine as a reducing agent) in the absence of an electron acceptor. The following substrates (10 mM each) were tested: fructose, fumarate, glucose, glycerol, lactate, malate, mannitol, pyruvate, succinate, and 0.3% wt/vol. yeast extract. Fermentation of substrates was defined as the ability to grow after three successive 10% transfers, and growth was monitored by measuring optical density at 600 nm.

### Analytical Techniques

AQDS and anthrahydroquinone-2,6-disulfonate (AH_2_DS) were measured spectrophotometrically at 405 and 325 nm under anoxic conditions. The molar absorptivity coefficients calculated for AQDS were 𝜀_405_ = 0.13 and 𝜀_325_ = 6.1 mM^-1^ cm^-1^; for AH_2_DS, the coefficients used were 𝜀_405_ = 10.3 and 𝜀_325_ = 0.8 mM^-1^ cm^-1^. Aqueous Fe(II) in sample filtrate (filtered through 0.2-μm filter) that was diluted 1:10 in 0.5 N HCl was quantified spectrophotometrically at 562 nm with ferrozine (Lovley and Phillips, 1987). Diphenylcarbazide reagent (Hach, Loveland, CO, United States) was used to quantify Cr(VI) at 540 nm. Ion chromatography (IC25 Ion Chromatograph Ion Pac-ATC-HC Trap Column) was used to measure NO_3_^-^, NO_2_^-^, SO_4_^2-^, and SeO_4_^2-^. Reduction of As(V) was evaluated by measuring soluble As(V) at 865 nm in samples preserved in KIO_3_ and HCl as described previously (Cummings et al., 1999).

X-ray photoelectron spectroscopy (XPS) was employed to determine the valence state of Fe in cell suspensions incubated with 5 mM AQDS and 2 line-ferrihydrite or ferrihydrite alone. The extent of Fe(III)-reduction at both uncolonized and colonized Fe(III)-oxide surfaces was examined. Ferrihydrite samples were dried onto a Si wafer before mounting for XPS analysis. Samples were mounted for XPS in an anaerobic glove box and transported to the spectrometer in a plate chamber sealed under an anaerobic atmosphere. Brief (<5 s) exposure to air occurred during introduction of the samples into a N_2_-flushed antechamber. This antechamber was then evacuated and the sample placed into the spectrometer itself. Spectra were collected on a Perkin–Elmer Physical Electronics Division Model 5600ci spectrometer (Perkin–Elmer Inc., Eden Prairie, MN, United States). The spectrometer was calibrated employing the Au 4f_7/2_, Cu 2p_3/2_, and Ag 3d_5/2_ photopeaks with binding energies of 83.99, 932.66, and 368.27 eV, respectively. A consistent 400-μm spot size was analyzed on all surfaces using a monochromatized Al Kα (hν = 1486.6 eV) X-ray source at 300 W and a pass energy of 46.95 eV for survey scans, and 11.75 eV for high-resolution scans. The system was operated at a base pressure of 10^-8^ to 10^-9^ torr. An emission angle (2ϕ) of 45° was used throughout. Following baseline subtraction (Shirley, 1972), curve-fitting was employed using an 80% Gaussian: 20% Lorentzian line shape to estimate the contributions of ferrous and ferric ions to the total Fe photopeak (described below). A common problem associated with the analysis of insulating materials such as Fe(III)-oxides is the accumulation of surface charge during spectral collection leading to photopeak shifts; this was overcome by the use of a 5-eV flood gun and by referencing of the principal C 1 s photopeak [nominally due to carbon of the type (–CH_2_–CH_2_–)_n_] to a binding energy (*E*_b_) of 284.8 eV (Swift, 1982).

Theoretical core *p* level models describing multiplet splitting associated with ferrous and ferric ions have been demonstrated by Gupta and Sen (Gupta and Sen, 1975) and empirical proofs substantiated by Pratt et al. (1994). Accordingly, ferric ion contributions were fit to high-resolution Fe 2p_3/2_ core regions with five peaks that decrease in intensity at increased E_b_, with all peaks having the same line-shape. Ferrous ion contributions are represented by a major peak accompanied by a pair of multiplet peaks occurring 0.9 eV to either side of the major peak and a shake-up satellite at elevated E_b_ [ΔE_b_∼6 eV, (McIntyre and Zetaurk, 1977)]. The relative intensities of the Fe(II) peaks are consistent with the aforementioned models and have been applied elsewhere in the identification of reduced iron in the presence of microorganisms (Herbert et al., 1998; Neal et al., 2001, 2004; Magnuson et al., 2004).

### DNA Base Composition and Cell Wall Analysis

Cells were grown overnight on R2A broth supplemented with 20 mM fumarate at 30°C and harvested by centrifugation. Cell pellets were suspended in a mixture of isopropanol and water (1:1). The G+C content of genomic DNA was determined by high-performance liquid chromatography using the method of Mesbah et al. (1989), and peptidoglycan was analyzed by the method of Schleifer and Kandler (1972). Both analyses were performed at Deutsche Sammlung von Mikroorganismen und Zellkulturen (DSMZ).

### DNA–DNA Hybridization

Cells of “*P. lilae*” UFO1 were grown in anoxic R2A broth at 30°C overnight and harvested by centrifugation. The pellet was suspended in a mixture of isopropanol and water (1:1) and submitted to DSMZ for DNA-DNA hybridization with *Pelosinus fermentans* R7^T^ (DSM 17108). DNA was isolated from the cell pellet using a French pressure cell and purified by chromatography on hydroxyapatite as described by Cashion et al. (1977). DNA-DNA hybridization was carried out as described previously (DeLey et al., 1970) with the modifications described by Huss et al. (1983) using a model Cary 100 Bio UV/VIS-spectrophotometer equipped with a Peltier-thermostatted 6 × 6 multicell changer and a temperature controller with *in situ* temperature probe (Varian). Percent DNA-DNA similarity was measured in 2X saline sodium citrate (SSC) solution at 65°C.

### Fatty Acid Methyl Ester Analysis

An overnight culture of “*P. lilae*” UFO1 was grown on R2A broth containing 20 mM fumarate at 30°C. The cells were harvested by centrifugation, frozen (-20°C), and analyzed by Microbial ID (Newark, DE, United States) for fatty acid methyl ester content.

### Electron Microscopy

Cells were grown overnight at 30°C in R2A broth containing 20 mM fumarate for morphological characterization using a Philips XL 30 Environmental Scanning Electron Microscope (ESEM), operating at 10 kV with a typical target current of 1.75 μA. Prior to imaging, the cells were washed twice and re-suspended in phosphate buffered saline (pH 7). Droplets of the washed cell suspension were placed in a conical ESEM mount to maximize solution volume. The chamber was maintained at ∼45% relative humidity (2.0°C, 2.4 torr of H_2_O) using a combination of Peltier cooling and differential pumping. ESEM imaging looked first at the edges of the mount then progressed inward to find regions of optimal cell density, minimal desiccation, and minimal precipitation of salts.

### 16S rRNA Gene Amplification

Genomic DNA was extracted from “*P. lilae*” UFO1 using the Mo Bio Ultra Clean Microbial DNA kit (Mo Bio Laboratories, Inc., Carlsbad, CA, United States). PCR amplification of the 16S rRNA gene was performed with primers 8F (5′-AGAGTTTGATCCTGGCTCAG-3′) (Eden et al., 1991), 907R (5′-CCGTCAATTCMTTTRAGTTT-3′) (Lane et al., 1985), 704F (5′-GTAGCGGTGAAATGCGTAGA-3′) (Lane et al., 1985), and 1492R (5′-GGTTACCTTGTTACGACTT-3′) (Lane, 1991). Each 50 μL PCR reaction mixture contained 1 U mL^-1^ of Deep Vent polymerase and 1X ThermoPol reaction buffer (New England Biolabs, Ipswich, MA, United States), 250 μM dNTPs, 800 nM of each primer, 2 mM MgSO_4_, and 1.5 μL of genomic DNA template. The PCR amplification conditions were as follows: an initial 95°C denaturation for 5 min, followed by 30 cycles of 95°C denaturation for 1 min, 56.1°C primer annealing for 1 min, and 72°C extension for 2.5 min, and then a final extension at 72°C for 4 min. PCR products were purified using the Qiagen QIAquick PCR Purification Kit (Valencia, CA, United States) per manufacturer’s instructions. The 16S rRNA gene PCR products were sequenced at the Idaho State University Molecular Research Core Facility (MRCF) on an ABI 3100 automated capillary sequencer (Applied Biosystems, Foster City, CA, United States) using 8F, 519R (5′-ATTACCGCGGCTGCTGG-3′) (Lane et al., 1985), 907R, 704F, 1100F (5′-CAACGAGCGCAACCCT-3′) (Lane et al., 1985), and 1492R primers in order to guarantee overlap of sequences. PCR products amplified with 8F and 1492R primers were also cloned in order to resolve the 3′ and 5′ ends of the 16S rRNA gene. Immediately following the PCR reaction, 3′-A overhangs were added to blunt-end 16S rRNA gene amplicons generated with 8F/1492R and Deep Vent polymerase using Taq polymerase (Invitrogen, Carlsbad, CA, United States) in an additional 10-min extension performed at 72°C. For addition of 3′-A overhangs, the 25 μL reaction contained the following: 16.2 μL of blunt-end PCR product, 1X buffer, 2 mM MgCl_2_, 400 μM dATP, and 1 U Taq polymerase. The resulting 16S rRNA gene product was cloned into the pCR4 ^®^-TOPO^®^ vector using the TOPO TA Cloning^®^ Kit for Sequencing (Invitrogen, Carlsbad, CA, United States) according to the manufacturer’s instructions. Plasmids were purified from clones using the Qiagen Qiaprep^®^ Spin Miniprep kit (Valencia, CA, United States) and sequenced directly using primers M13F (5′-GTAAAACGACGGCAG-3′), M13R (5′-CAGGAAACAGCTATGAC-3′), T3 (5′-ATTAACCCTCACTAAAGGGA-3′), and T7 (5′-TAATACGACTCACTATAGGG-3′) at the ISU MRCF. The GenBank accession number for the 16S rRNA sequence of “*P. lilae*” UFO1 is DQ295866, and the draft genome accession number is CP008852.

### Phylogenetic Analysis

Species representing the Negativicutes Class were determined based on the List of Prokaryotic Names with Standing in Nomenclature (LPSN)^1^ (Parte, 2014). Phylogenetic analysis was conducted using both ARB, version 6.0.6 (Strunk et al., 1996) software, and the SILVA sequence database SSURef_NR99_128_SILVA_07_09_16_opt^2^ (Quast et al., 2013). Sequences not available in this SILVA database were downloaded from GenBank^3^. SILVA sequence alignments were used with a few refinements made manually. Sequences were filtered using SILVA’s positional variability by parsimony filter for bacteria (pos_var_ssuref:bacteria). Phylogenetic tree was inferred within the ARB software by the Maximum Likelihood method PHYML, version 20130708 (Guindon and Gascuel, 2003), using nucleotide substitution model HKY85 (Hasegawa et al., 1985) and branch supports determined by Bayesian estimation.

## Results and Discussion

### Strain Isolation and Morphology

**FIGURE 1:**
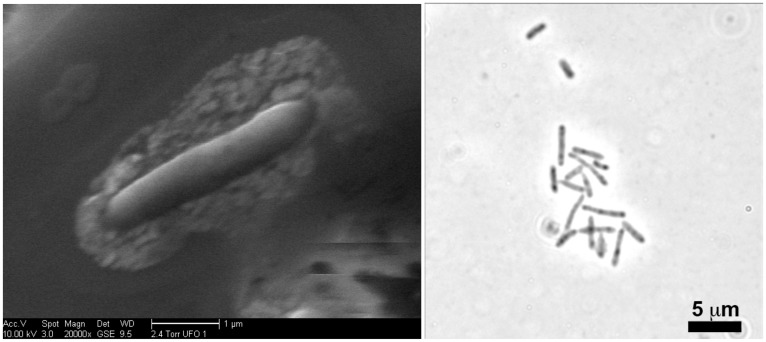
Left Panel: Scanning electron micrographs revealing extracellular materials surrounding a cell of “*P. lilae*” UFO1. Right Panel: Light micrograph of a culture of “*P. lilae*” UFO1 with apparent spore-like structures, the dark areas within cells, usually located at the cell terminus. Note the extracellular materials associated with the cell.

“*Pelosinus lilae*” UFO1 was isolated under strictly anaerobic conditions from dilution-to-extinction cultivation experiments, and was capable of AQDS reduction, as evident by a color change in growth medium from transparent, pale yellow to bright orange. An aliquot of enrichment culture was streaked on R2A plates containing 20 mM fumarate, and after 48–72 h of anaerobic incubation, small, round, white colonies were apparent. After re-streaking at least three times, a colony was transferred to FW medium containing lactate and AQDS, and the culture reduced AQDS. “*P. lilae*” UFO1 is a strict anaerobe with a fermentative metabolism, and microscopic observations revealed that it is rod-shaped, 0.2–0.7 × 1.5–4.7 μm in size (**Figure 1**-left panel), motile, and stains Gram-variable. Structures that appeared to be spores were also visible (**Figure 1**-right panel). Extracellular polymeric substance (EPS) is associated with individual cells, as judged by electron-dense materials surrounding the cell periphery. The production of spores was supported by the observation that a 10% inoculum from thermally-treated (85°C for 32 min) cell suspensions of “*P. lilae*” UFO1 could be re-grown in R2A broth and also exhibited the ability to reduce AQDS in the presence of H_2_ upon transfer to fresh media.

### Temperature and pH Tolerance

**FIGURE 2:**
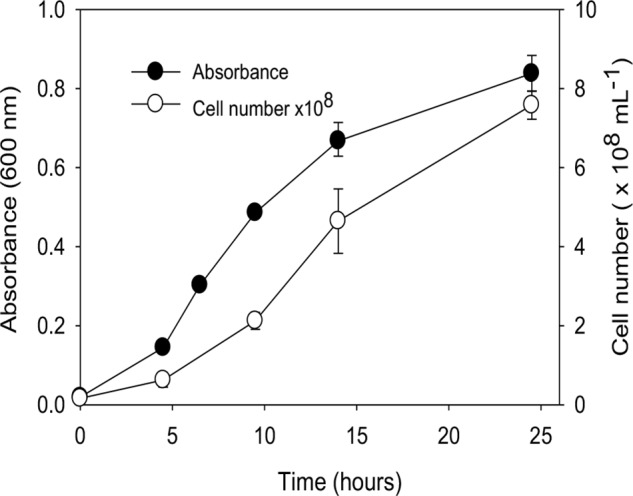
Growth curve of “*P. lilae*” UFO1 on R2A broth supplemented with 20 mM fumarate at 30°C. Symbols are means of triplicate analyses, and error bars indicate ± 1 standard deviation.

A growth curve for “*P. lilae*” UFO1 at 30**°**C is shown in **Figure 2**. Optimal temperature and pH for growth of “*P. lilae*” UFO1 were determined using R2A broth supplemented with 20 mM fumarate. The range of growth temperatures was 22–37°C, with an optimum of 37°C; average generation times at 22, 30, and 37°C were 4.6, 2.9, and 1.9 h, respectively. The range of pH for growth on R2A broth with 20 mM fumarate was 5.5–8, with optimum growth at pH 7. No growth was observed at temperatures of 13°C or 42°C, or at pH ≤ 5.4 or pH ≥ 8.5.

### Electron Donor Utilization

Carbon sources utilized in Biolog AN microplates were D-fructose, L-fucose, D-galactose, D-galacturonic acid, D-glucose-6-phosphate, glycerol, 3-methyl-D-glucose, palatinose, L-rhamnose, α-ketobutyric acid, α-ketovaleric acid, pyruvic acid, and succinic acid. “*P. lilae*” UFO1 fermented the following substrates (as evidenced by growth in the absence of an externally supplied electron acceptor): fructose, fumarate, glucose, glycerol, lactate, mannitol, pyruvate, and yeast extract. Malate and succinate were tested but not fermented. “*P. lilae*” UFO1 did not grow chemoautotrophically on H_2_-CO_2_ (see **Table 1**).

**Table 1:** Reduction of 5 mM AQDS with different electron donors.

Electron donor	AH_2_DS (mM)
Acetate (10 mM)	0.03 ± 0.00
Benzoate (0.5 mM)	0.04 ± 0.01
Ethanol (10 mM)	0.04 ± 0.01
Formate (10 mM)	0.05 ± 0.01
Glucose (5 mM)	1.05 ± 0.01
Glycerol (10mM)	1.01 ± 0.48
H_2_ (5 ml in headspace)	2.52 ± 0.17
Phenol (0.5 mM)	0.04 ± 0.01
Propionate (10 mM)	0.05 ± 0.01
Pyruvate (10 mM)	1.47 ± 0.03
Succinate (10 mM)	0.22 ± 0.10
Yeast Extract (0.25%)	2.20 ± 0.10

*Average production of AH_2_DS in triplicate cultures (±1 standard deviation) after 7 days of incubation at 30°C with “P. lilae” UFO1.*

### Metal Transformation

Although “*P. lilae*” UFO1 was isolated from an Fe(III)-reducing enrichment by cultivation with acetate and AQDS, this “*P. lilae*” was not capable of respiratory growth on AQDS when 10 mM acetate was provided as the electron donor. AQDS was reduced to AH_2_DS only in the presence of fermentable substrates or H_2_ (**Table 1**). In the presence of 10 mM lactate, nitrate was reduced to nitrite following 1 week of incubation. As(V), NO_3_^-^, SeO_4_^2-^, and SO_4_^2-^ were not reduced in the presence of 10 mM lactate.

**FIGURE 3:**
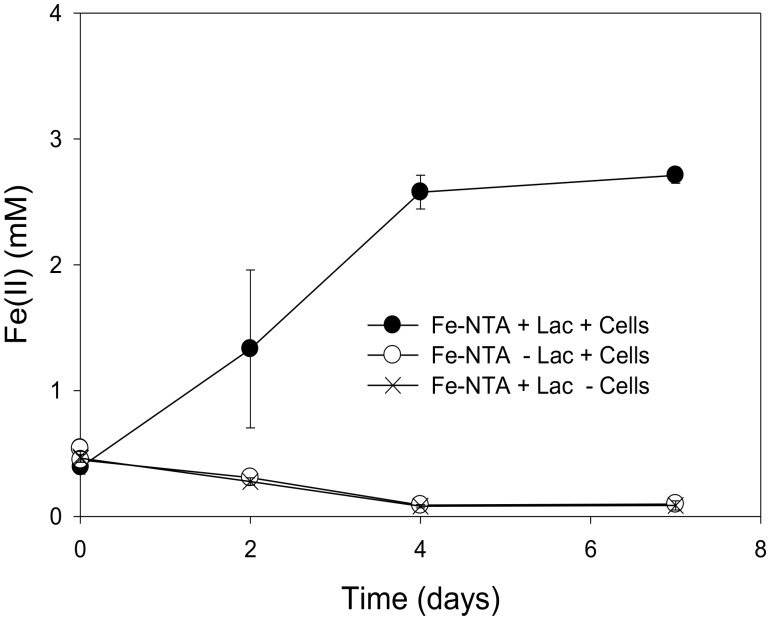
Incomplete reduction of Fe(III)-NTA (10 mM) by “*P. lilae*” UFO1 in the presence of a fermentable substrate, 10 mM lactate after 7 days of incubation. Symbols are means of triplicate cultures, and error bars indicate ± 1 standard deviation.

The incomplete reduction of 10 mM Fe(III)-NTA by “*P. lilae*” UFO1 is shown in **Figure 3**. In the presence of 10 mM lactate, nearly 3 mM Fe(II) was produced after 1 week of incubation. Production of Fe(II) was not evident in abiotic controls. Biotic reduction of Fe(III)-NTA in the absence of lactate did not occur.

High-resolution core Fe 2p_3/2_ spectra collected on a cell-free control containing ferrihydrite incubated with culture medium and H_2_ suggested that abiotic Fe(III)-reduction did not take place under these conditions (**Table 2**). The collected peak envelope is described well by the Fe(III) multiplet splitting model and is in agreement with published iron oxide spectra (data not shown). However, cell-free controls incubated with H_2_ + AQDS, lactate, or lactate + AQDS revealed the presence of 14.8, 26.2, and 26.0 atom% Fe(II), respectively, indicating some abiotic reduction of ferrihydrite occurred. Incubation of ferrihydrite with strain UFO1 and H_2_, both with and without AQDS, revealed a significant increase in the Fe(II) contribution compared to the abiotic controls. Addition of a principal Fe(II) peak at 708.4 eV for the ferrihydrite spectra (together with associated multiplet and satellite peaks) was required to complete the model (not shown). In contrast, lactate did not appear to significantly enhance reduction of ferrihydrite by “*P. lilae*” UFO1 regardless of the presence of AQDS.

**Table 2:** Results of fitting multiplet splitting peak models to X-ray photoelectron spectroscopy–derived Fe 2p_3/2_ photopeaks collected from 2-line ferrihydrite recovered from cultures of UFO1 in the presence and absence of AQDS with H_2_ and lactate compared to abiotic controls.

Treatment	Atomic % abiotic	Atomic % biotic
HFO + H_2_	0.0	14.5
HFO + H_2_ + AQDS	14.8	54.4
HFO + Lactate	26.2	31.2
HFO + Lactate + AQDS	26.0	33.4

**FIGURE 4:**
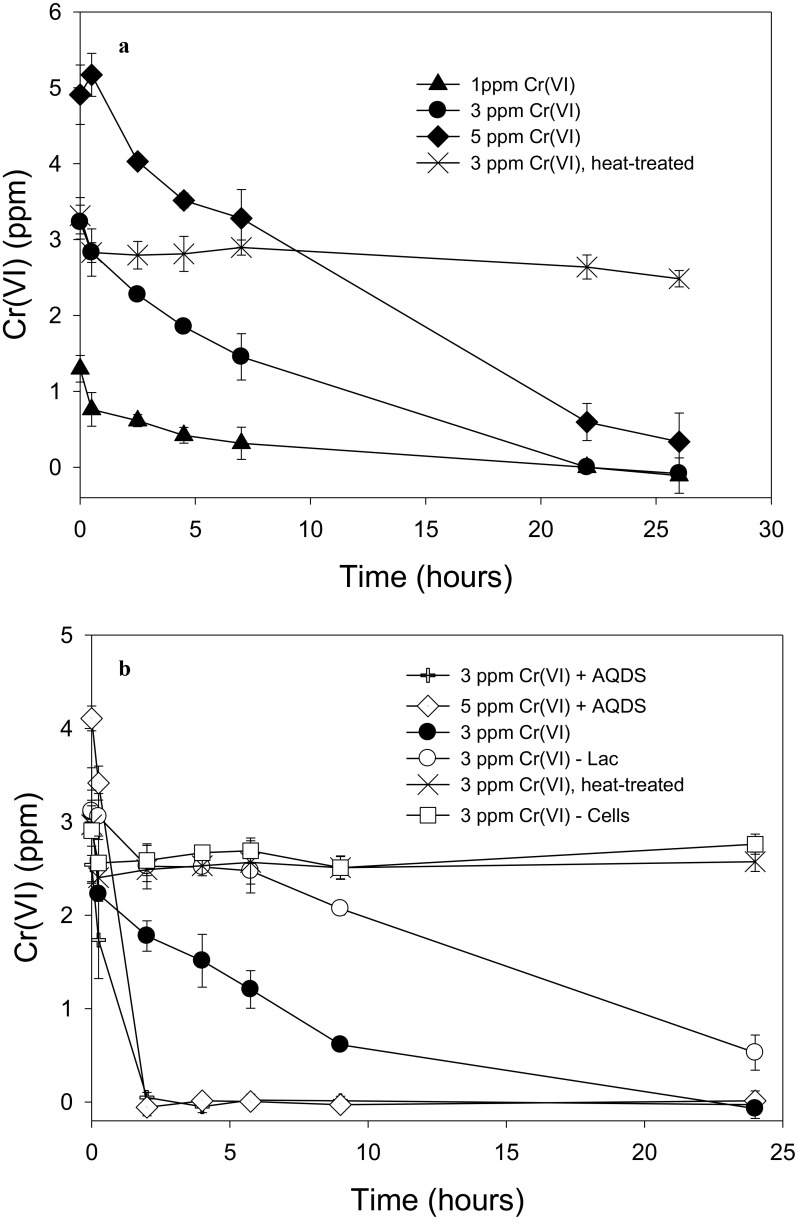
**(A)** Removal of 1, 3, and 5 ppm Cr(VI) by “*P. lilae*” UFO1. Lactate (10 mM) was present in all treatments. **(B)** Cr(VI) removal in the presence or absence of 1 mM AQDS, and removal of Cr(VI) in lactate-free controls. Lactate (10 mM) was present in all treatments unless otherwise noted. Symbols are means of triplicate analyses, and error bars indicate ± 1 standard deviation.

Soluble Cr(VI) was removed from solution in cell suspensions of “*P. lilae*” UFO1 under non-growth conditions. **Figure 4A** shows the nearly complete removal of 1 and 3 ppm Cr(VI) by strain UFO1 within 24 h when lactate (10 mM) was provided as an electron donor, whereas 5 ppm Cr(VI) decreased to an average 0.3 ppm in 26 h. Over the course of the experiment, controls containing heat-treated cells (90°C, 32 min) did not exhibit significant Cr(VI) removal suggesting that sorption to cell surfaces did not contribute to the removal of Cr(VI) seen in other treatments. In **Figure 4B**, it is evident that Cr(VI) removal was dramatically enhanced in the presence of 1 mM AQDS. For treatments containing 3 and 5 ppm Cr(VI) with lactate (10 mM) plus 1 mM AQDS, Cr(VI) concentrations dropped to less than 0.1 ppm after just 2 h of incubation with cell suspensions of strain UFO1. There was no significant change in Cr(VI) concentrations in cell-free and heat-treated controls. In contrast to the Fe(III)-NTA results, where lactate appeared to be required for Fe(III) reduction, Cr(VI) was removed in the absence of lactate from 3 to 0.5 ppm in 24 h, which may suggest the use of endogenous carbon reserves by “*P. lilae*” UFO1, if removal is assumed to be the result of reduction. Others have similarly observed removal of soluble Cr(VI) by other bacterial species in the absence of exogenous electron donors (Ishibashi et al., 1990; Shen and Wang, 1993; Sani et al., 2002), suggesting that Cr(VI) removal occurs via the action of an enzyme utilizing endogenous electron donors (i.e., cellular NADH, FADH_2_) with a non-specific NADH oxidoreductase activity that can reduce Cr(VI) to Cr(III). In *Pelosinus* sp. HCF1 (Beller et al., 2013), Cr reduction was thought to be linked to a flavoprotein related to the ChrR family of chromate reductases, with potential involvement of hydrogenases. Genome analysis suggests a similar potential in “*P. lilae*” UFO1 (see below).

“*P. lilae*” UFO1 was previously evaluated for its U(VI) transformation abilities (Ray et al., 2011), and a potential mechanism for U(VI) sequestration in this isolate was discovered (Thorgersen et al., 2017). Interactions of U(VI) with extracellular materials led to the discovery of S-layer mediated binding of U(VI), as well as some reduction activity [appearance of U(IV)]. While the precise mechanisms of sequestration and reduction of Cr, U, and Fe in strain UFO1 remain poorly characterized, we propose that a combined sequestration/transformation mechanism is at play for dealing with Cr, U, Fe, and perhaps a variety of other metals not yet evaluated. Previous studies examining S-layer metal binding in *Bacillus* support this idea (Velásquez and Dussan, 2009), but the true functionality of “*P. lilae*” UFO1 S-layer proteins in toxic metal binding and sequestration remains to be investigated.

### Biochemical Characteristics

The majority of fatty acids identified in strain UFO1 were straight unsaturated chains in *cis* conformation (**Table 3**). The predominant fatty acids identified were C_15:1_ ω8c, C_17:1_ ω8c, and C_15:0_. “*P. lilae*” UFO1 had straight saturated (35.52%) and unsaturated (62.38%) chains, 6.59% C_11_ and C_13_ fatty acids, 74.87% C_15_ and C_17_. The fatty acid profile of strain UFO1 is consistent with profiles characteristic of members of the Class Negativicutes described previously (Strompl et al., 1999) with the exception of 3-hydroxy fatty acids, which were only present at 1.82%. Dimethyl acetals (C_14:0_ DMA and C_14:1_ ω7c DMA) accounted for 9.52% of the total fatty acids identified and are characteristic of anaerobic bacteria. Significant amounts of dimethyl acetals among members of the Class Negativicutes have been reported previously (Moore et al., 1994).

**Table 3:** Equivalent chain length (ECL) and fatty acid composition (%) of “*P. lilae*” UFO1 and comparator strains.

		1	2	3

**ECL**	**Fatty Acid**	**%**	**%**	**%**
9.00	C_9:0_	0.79	1.7	1.4
10.00	C_10:0_	0.28	1.8	2.8
10.61	i-C_11:0_	1.25	4.0	3.6
11.00	C_11:0_	2.82	3.6	4.9
13.00	C_13:0_	1.67	0.5	1.0
13.46	C_12:0_ 3OH	0.97	ND	ND
14.00	C_14:0_	0.72	0.7	1.9
14.11	i-C_13:0_ 3OH	0.85	4.6	1.2
14.28	C_14:1_ ω7c DMA	1.27	1.3	0.9
14.47	C_14:0_ DMA	8.25	10.0	12.4
14.79	C_15:1_ ω8c	28.45	ND	ND
14.85	C_15:1_ ω6c	0.99	1.4	0.8
15.00	C_15:0_	13.45	5.9	5.5
15.77	C_16:1_ ω9c	2.04	2.4	4.7
15.81	C_16:1_ ω7c	1.09	2.3	2.2
16.00	C_16:0_	1.92	1.3	1.9
16.79	C_17:1_ ω8c	26.52	ND	ND
16.86	C_17:1_ ω6c	1.53	1.2	0.7
17.00	C_17:0_	3.93	1.3	0.5
17.77	C_18:1_ ω9c	0.49	0.3	0.9
18.00	C_18:0_	0.72	ND	ND

*1 Pelosinus strain UFO1, 2 Pelosinus defluvii SHI-1^T^ 3 Pelosinus fermentans DSM 17108^T^.*

The cell wall of “*P. lilae*” UFO1 contained meso-diaminopimelic acid (m-Dpm) as the diagnostic diamino acid in the total hydrolysate of the peptidoglycan. Alanine and glutamic acid were also present in the peptidoglycan. Partial hydrolysis of the peptidoglycan revealed the presence of the peptides L-ala—D-glu and Dpm—D-ala. From these data, it was concluded that “*P. lilae*” UFO1 shows the directly cross-linked peptidoglycan type, A1γ m-Dpm-direct (Schleifer and Kandler, 1972).

The G+C content of the genomic DNA of “*P. lilae*” UFO1 was 38.0 mol% (**Table 4**). The DNA G+C content of *P. fermentans* R7^T^, is 41.0 mol% (Shelobolina et al., 2007). Additionally, duplicate DNA-DNA hybridizations conducted with strain UFO1 against *P. fermentans* R7^T^ showed 9.8 and 23.7% DNA-DNA similarity, indicating that strain UFO1 does not belong to the species *P. fermentans* as defined by the threshold value of 70% DNA-DNA relatedness (Wayne et al., 1987).

**Table 4:** Distinguishing features of strain UFO1 compared to the most closely related described species in the Class Negativicutes.

Trait	1	2	3	4	5	6	8
G+C content of DNA (mol %)	38.0	41.0	ND	51.5	52.0−54.0	35.0	39.2
Cell shape	Slightly curved or straight rods	Straight rods	Straight rods	Straight rods	Straight rods	Curved rods	Rods
Spore formation	+	+	+	+	+	−	ND
Motility	+	+	+	+	+	+^∗^	ND
Cell size (μm)	0.2−0.7 × 1.5−4.7	0.6 × 2−6	0.5−0.7 × 2.2−12	0.6 × 6−60	0.5 × 3	0.5 × 2−10	1 × 2−7
Temperature range (°C)	22−37	4−36	19−35	19−40	20−45	10−42	10−42
Temperature optimum (°C)	37	22−30	30	30−33	25−30	37	
pH range	5.5−8	5.5−8	6.2−8.2	6.4−8.6	ND	5−8.5	7.0−7.5
pH optimum	7	7	7.8	7.8	ND	6.5−7.5	7.0
Growth on:					
Fructose	+	+	+	+	+	−	ND
Fumarate	+	+	+	+	ND	−	+
Glucose	+	+	+	+	ND	−	ND
Glycerol	+	−	+	−	+	+	+
H_2_+CO_2_	−	−	−	+	−	−	−
Lactate	+	+	+	−	ND	−	ND
Malate	−	+	ND	−	ND	−	ND
Mannitol	+	+	ND	+	−	−	−
Pyruvate	+	+	+	+	ND	−	ND
Succinate	−	+	−	−	ND	−	ND

*1-Strain UFO1 (this study); 2 Pelosinus fermentans R7^T^ (Shelobolina et al., 2007); 3 Pelosinus propionicus TmPN3 (Boga et al., 2007; Moe et al., 2012); 4 Acetonema longum APO-1^T^ (Kane and Breznak, 1991); 5 Dendrosporobacter quercicolus DSM 1736^T^ (Stankewich et al., 1971; Strompl et al., 2000); 6 Anaerosinus glycerini strain LGS 4^T^ (Schauder and Schink, 1989; Strompl et al., 1999); 8 Pelosinus defluvii SHI-1^T^ (Moe et al., 2012). ^∗^motility lost after 1 year of cultivation. ND, not determined.*

### 16S rRNA Gene Sequence and Phylogenetic Analysis

**FIGURE 5:**
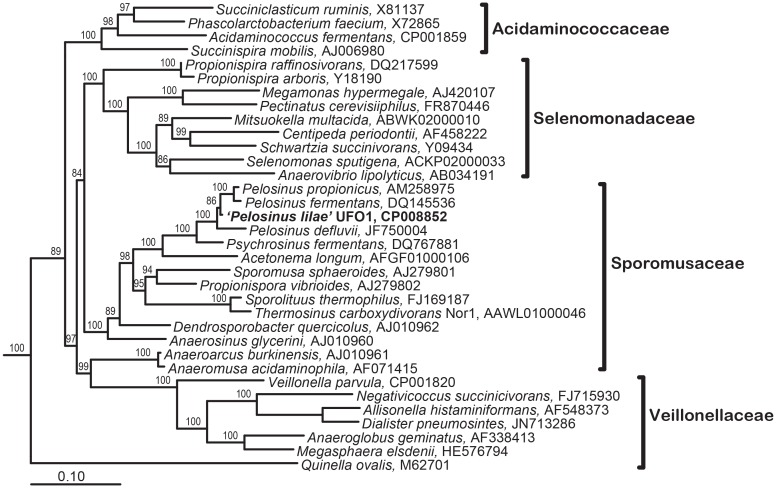
Maximum Likelihood tree showing phylogenetic relationships of the 16S rRNA genes in the Class Negativicutes; the types species for each genus in the four families within this class is included. Branch supports determined by Bayesian estimation ≥80 are shown at branch points. Scale bar indicates 0.1 changes per nucleotide. Tree was rooted with 4 species (not shown): *Bacillus subtilis* (AB042061), *Clostridium acetobutylicum* (AE001437), *Desulfotomaculum acetoxidans* (Y11566), and *Heliobacillus mobilis* (AB100835).

“*Pelosinus lilae*” UFO1 is firmly included within the *Pelosinus* clade in the *Sporomusaceae* family with branch support of 100 (**Figure 5**). Results indicate that “*P. lilae*” UFO1 is phylogenetically distinct from the most closely related organisms *P. fermentans* R7^T^ (Shelobolina et al., 2007), *Pelosinus propionicus* strains TmPN3^T^and TmPM3 (originally published as *Sporotalea propionica*) (Boga et al., 2007), and *Pelosinus defluvii* (Moe et al., 2012). The distances between 16S sequences from “*P. lilae*” UFO1 and the closest type strains, *P. fermentans* strain R7^T^ and *P. propionicus* TmPN3^T^, were 1.84 and 2.40% respectively, which are greater than the distance between the described type strains, 0.9%. A BLAST search of the 16S rRNA gene sequence of strain UFO1 revealed 99% similarity (over 1041 nucleotide bases) with a clone detected in a pH 5, Fe(III)-reducing enrichment established with background sediments from the FRC, pH5lac302-37 (AY527741) (Petrie et al., 2003). Additionally, two clones detected by Petrie and co-workers (Petrie et al., 2003) that were from Fe(III)-reducing enrichments established with contaminated FRC sediments also shared a high degree of 16S rRNA gene sequence similarity with “*P. lilae*” UFO1: 97% for Gly030-8A (AY524569) and 97% for Gly030-5C (AY524568). A recent paper by Newsome et al. (2015) report the presence of *Pelosinus* in enrichments of sediments from a United Kingdom nuclear site. Stimulation with glycerol phosphate resulted in substantial increases in bacteria closely related to *Pelosinus*, which comprised 33% of bacteria identified at the genus level. This work implicates *Pelosinus* species as having a key role in the removal of soluble U(VI) via precipitation to a reduced, crystalline U(IV) phosphate mineral, considered to be more recalcitrant to oxidative remobilization, in contaminated sediments.

During the analysis of the 16S rRNA gene sequence of “*P. lilae*” UFO1, significant inter-operon heterogeneity among 16S rRNA gene clones from “*P. lilae*” UFO1 was detected. Sequencing of cloned 16S rRNA gene PCR products revealed two distinct 16S rRNA gene sequences were present in strain UFO1. These differences were not due to the presence of a contaminant, as the culture was extensively purified prior to analysis (Ray et al., 2010). In one group of clones a 100-bp insertion was present near the 5′ end of the sequence. This type of 16S rRNA gene sequence heterogeneity was previously reported for the, proposed but not validly published, member of the Class Negativicutes “*Anaerospora hongkongensis*” (Woo et al., 2005; Beller et al., 2013). 16S rRNA length heterogeneity has also been reported in a few other unrelated species, notably *Paenibacillus polymyxa* (Nubel et al., 1996), *Desulfotomaculum kuznetsovii* (Tourova et al., 2001), *Aeromonas* strains (Morandi et al., 2005), *Bacillus clausii* (Kageyama et al., 2007), and the archaeon *Haloarcula marismortui* (Mylvaganam and Dennis, 1992; Amann et al., 2000). Further work has demonstrated that the insert-bearing 16S rRNA gene sequence was not functional in the ribosomes of strain UFO1 (Ray et al., 2010).

### Genome Annotation and Implications for Metal Transformation

The draft genome sequence for strain UFO1 (Brown et al., 2014) was data-mined for genes potentially involved in metal transformation. Pathways relevant to metal transformation include 2 loci encoding arsenate reductase (UFO1_2328, UFO1_2536), subunits for a cytochrome c-dependent nitrate reductase (UFO1_1541, UFO1_1544), and several loci for flavin reductases (thought to be involved in chromium transformation). A predicted Co-Zn-Cd efflux system (UFO1_2233) is also present. Additionally, “*P. lilae*” UFO1 has a *chrA* locus for chromate transport (UFO1_3236), and a NiFe-hydrogenase (UFO1_2674) linked to a cytochrome *b* (UFO1_2673). Obviously, characterized metal-reducing Firmicutes, including strain UFO1, harbor a multiplicity of genes conferring metal detoxification ability, further extending the importance of these organisms in the biogeochemical processing of toxic metals.

### Potential Ecophysiologic Role of “*Pelosinus lilae*” UFO1 in Subsurface Environments

“*Pelosinus lilae*” UFO1 represents a novel addition to a recently described and poorly characterized genus of fermentative bacteria. The most closely-related species, *P. fermentans* R7^T^, was similarly isolated from an Fe(III)-reducing enrichment; however, the enrichment was established with kaolin lenses originating from Plast, Russia (Shelobolina et al., 2007). This suggests that representatives of *Pelosinus* may be widespread in anoxic environmental systems. Kappler et al. (2004) demonstrated that fermenting bacteria were one of the largest populations of bacteria in freshwater lake sediments and had an important role in humic acid reduction; the results suggested that humic acid-mediated reduction of poorly soluble Fe(III) oxides is an important reductive pathway in anoxic natural environments. “*P. lilae*” UFO1 demonstrated the ability to reduce the humic acid analog, AQDS, in the presence of H_2_ and fermentable substrates; in addition, AQDS mediated the reduction of the insoluble Fe(III)-oxide, ferrihydrite. The presence of AQDS also enhanced Cr(VI) removal from solution. The AQDS-AH_2_DS couple has a standard potential (E^o^) of -184 mV at pH 7 (Fultz and Durst, 1982; Wolf et al., 2009), which is well below that for the CrO_4_^2-^-Cr(OH)_3_ couple (+480 mV at pH 7) (Takeno, 2005); therefore, the transfer of electrons from AH_2_DS to CrO_4_^2-^ is thermodynamically favorable and suggests Cr(VI) removal in the presence of AQDS was likely due to reduction. These findings suggest that fermentative bacteria such as strain UFO1 may play a role in the reduction of humic acids, which may in turn facilitate the reduction of metallic contaminants in subsurface environments.

*“Pelosinus lilae*” UFO1 was isolated from pristine sediments beneath Oak Ridge National Laboratory, Oak Ridge, TN, United States. Organisms with high 16S rRNA gene sequence similarity to strain UFO1 have been detected in Fe(III)-reducing (Petrie et al., 2003) and U(VI)-reducing (Nyman et al., 2007) enrichments initiated with contaminated sediments, which may suggest the prevalence of *Pelosinus* species in the subsurface. Strain UFO1, and organisms with similar metabolic capabilities also offer potential for the removal of soluble U(VI) from cell suspensions (Ray et al., 2011). “*P. lilae*” UFO1 has been shown to reduce metals with or without an exogenous “electron shuttle” such as AQDS, and genome analysis predicts additional metal-transformation pathways that have not yet been verified. The transformation of an array of electron acceptors such as AQDS, Fe(III), Cr(VI) and U(VI) by UFO1 suggest a potentially important role for this and similar organisms in influencing the biogeochemistry of pristine and contaminated geologic media at the ORFRC.

## Author Contributions

AR, SC, AN, JI, and DC conducted the experiments. AR, SC, YF, DC, and TM designed the experiments. All authors contributed to the preparation of the manuscript.

## Conflict of Interest Statement

The authors declare that the research was conducted in the absence of any commercial or financial relationships that could be construed as a potential conflict of interest.

## Footnotes

1 www.bacterio.net

2 www.arb-silva.de

3 www.ncbi.nlm.nih.gov
